# Design, efficient synthesis and molecular docking of some novel thiazolyl-pyrazole derivatives as anticancer agents

**DOI:** 10.1186/s13065-019-0632-5

**Published:** 2019-09-24

**Authors:** Abdelwahed R. Sayed, Sobhi M. Gomha, Fathy M. Abdelrazek, Mohamed S. Farghaly, Shaimaa A. Hassan, Peter Metz

**Affiliations:** 1 Department of Chemistry, Faculty of Science, King Faisal University, Hofuf, Saudi Arabia; 2 Chemistry Department, Faculty of Science, Cairo University, Giza, 12613 Egypt; 3 Department of Chemistry, Faculty of Science, Beni-Suef University, Beni-Suef, Egypt; 4 Department of Chemistry, Faculty of Science, Islamic University in Almadinah Almonawara, Almadinah Almonawara, 42351 Saudi Arabia; 5 Science and Technology Center of Excellence, Ministry of Military Production, Cairo, Egypt; 6 Fakultat Chemie und Lebensmittelchemie, TU-Dresden, 01069 Dresden, Germany

**Keywords:** Coupling reaction, Cyclocondensation, Hydrazonoyl halides, Pyrazolones, Anticancer activity, Molecular docking

## Abstract

Pyrazoles, thiazoles and fused thiazoles have been reported to possess many biological activities. 3-Methyl-5-oxo-4-(2-arylhydrazono)-4,5-dihydro-1*H*-pyrazole-1-carbothioamides **3a**,**b** (obtained from the reaction of ethyl 3-oxo-2-(2-arylhydrazono)butanoates **1a**,**b** with thiosemicarbazide) could be transformed into a variety of thiazolyl-pyrazole derivatives **6a**–**h**, **10a**–**c**, **15a**–**c**, **17**, **19** and **21** via their reaction with a diversity hydrazonoyl chlorides as well as bromoacetyl derivatives. Moreover, the computational studies were carried out for all new compounds. The results indicated that five compounds showed promising binding affinities **(10a**: − 3.4 kcal/mol, **6d**: − 3.0 kcal/mol, **15a**: − 2.2 kcal/mol, **3a**: − 1.6 kcal/mol, and **21**: − 1.3 kcal/mol) against the active site of the epidermal growth factor receptor kinase (EGFR). The cytotoxicity of the potent products **3a**, **6d**, **10a**, **15a**, and **21** was examined against human liver carcinoma cell line (HepG-2) and revealed activities close to Doxorubicin standard drug. There was an understanding between the benefits of restricting affinities and the data obtained from the practical anticancer screening of the tested compounds.
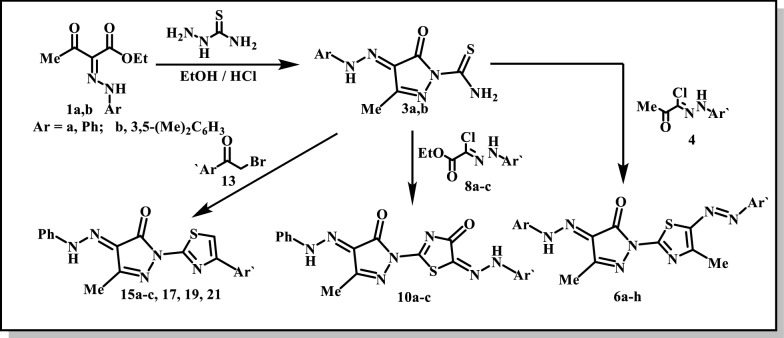

## Introduction

The pyrazole nucleus has many biological activities, including several pharmaceuticals currently on the market. Moreover, pyrazole derivatives have found numerous applications in fluorescent substances, dyes, agrochemicals, and more. Therefore, the interest in pyrazole chemistry is still ongoing [1–6]. Thiazoles are additionally significant class of heterocyclic compounds, found in numerous powerful biologically active drugs such as Ritonavir (antiretroviral drug), Sulfathiazole (antimicrobial drug), Tiazofurin (antineoplastic drug), and Abafungin (antifungal drug) [7]. Compounds containing thiazole show many biological activities such as antihypertensive, antimicrobial and antifungal, anti-HIV, anticonvulsant and anti-inflammatory activities [8–12].

Thiazole derivatives are also known to possess several anticancer activities [13–15]. There are several mechanisms for the antitumor action of thiazole derivatives, acting on cancer biotargets, such as inosine monophosphate dehydrogenase (IMPDH) [16], tumor necrosis factor TNF-α [17] and apoptosis inducers. The biological profiles of these new generations of thiazole would represent a productive matrix for further advancement of better anticancer specialists. Drug design part guarantees that thiazole is best particle for the said target activity. Thiazoles have better action as an anticancer just as it demonstrates better binding domain and they have less cytotoxicity to normal cell (physiological cell) however alongside that it has site explicit movement to malignant growth cell (pathological cell). We can seek after the superior to best treatment for malignant growth treatment since it limit side and unfriendly impact and it additionally indicates target oriented action.

**Fig. 1:**
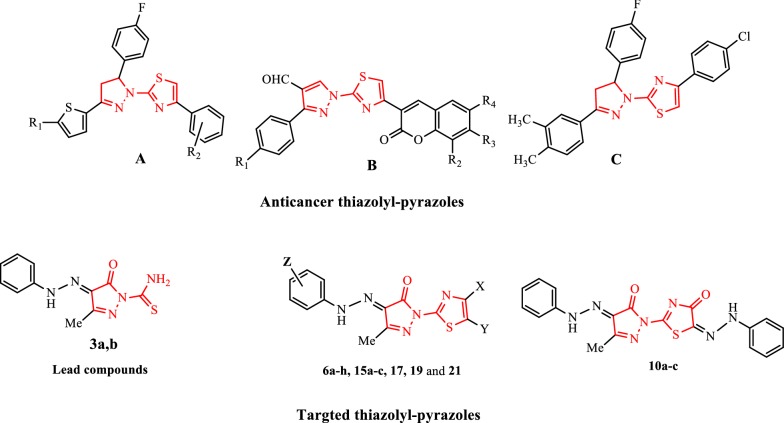
Anticancer activity of some reported thiazolyl-pyrazoles **a**–**c** and the target compounds

Thiazolyl-pyrazole hybrids have displayed antitubercular [18, 19], anti-inflammatory, antimicrobial [20], antimycobacterial activities [21], and FabH inhibitors [22]. Moreover, thiazolyl-pyrazole compounds were identified as as potential anticancer agents [23, 24] (Fig. 1). The EGFR PTKs have been identified as interesting targets for medicinal chemistry programs especially in cancer therapy [25]. Compounds that inhibit the kinase activity of EGFR after binding its cognate ligand are of potential interest as new therapeutic antitumor agents [26]. Lv et al. [27] reported that thiazolyl-pyrazole analogues showed modest to potent EGFR TK inhibitory and potential anticancer activities. The molecular docking results indicated that thiazolyl-pyrazolines were nicely bound to the EGFR kinase. Over the most recent two decades we have been associated with a program planning to orchestrate practically substituted heterocyclic compounds with foreseen biological activities that can be utilized as biodegradable agrochemicals from shoddy research facility accessible beginning materials [28–37]. In the edge of this program, it appeared important to synthesize a new class of compounds amassing the thiazole and the pyrazole moieties in one entity that may result in upgraded biological activity because of the synergistic impact of the two rings. A β-Ketoester seemed suitable starting material to fulfill this objective (Fig. 1).

## Results and discussion

### Chemistry

**Scheme 1:**
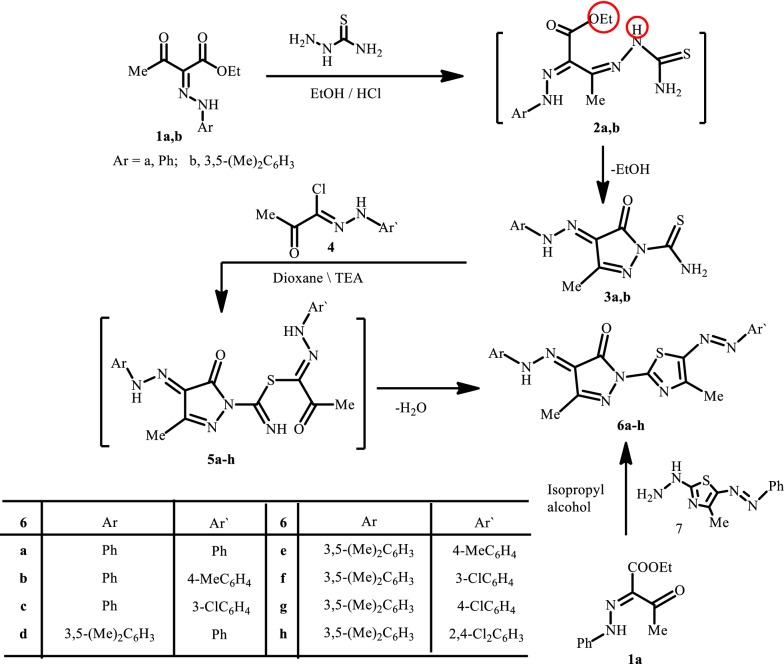
Synthesis of arylazothiazole derivatives **6a**–**h**

In the present work we used ethyl acetoacetate. To avoid interference of the active methylene in the following reactions ethyl acetoacetate was coupled with aryl diazonium salts to afford the hydrazo derivatives **1a**,**b**; which are the starting compounds for the synthesis of target thiazolyl-pyrazole derivatives. Thus, compounds **1a**,**b** were allowed to react with thisemicarbazide to afford 3-methyl-4-oxo-4-(2-arylhydrazono)-4,5-dihydro-1*H*-pyrazole-1-carbothioamides **3a**,**b** presumably via the intermediacy of the thiosemicarbazones **2a**,**b** which eliminate ethanol (Scheme 1).

The pyrazole derivatives **3a**,**b** react with the hydrazonoyl chlorides **4** to afford assumingly the thiohydrazonate intermediates **5a**–**h** which lose water to afford the final isolable thiazolyl-pyrazole derivatives **6a**–**h**. Spectral and analytical data of these compounds are in complete agreement with their proposed structures (*cf*. Scheme 1 and “Experimental”). A further evidence of the structure was deduced when the hydrazone **1a** reacted with the thiazolyl hydrazine **7** to afford a product which was found to be typically identical to **6a**.

**Scheme 2:**
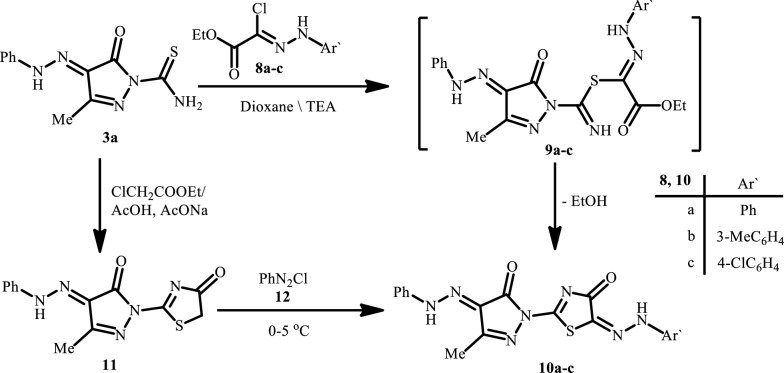
Synthesis of arylhydrazothiazole derivatives **10a**–**c**

The pyrazole derivative **3a** reacts with the hydrazonoyl chloride esters **8a**–**c** to afford the thiazolyl- pyrazole derivatives **10a**–**c** via the thiohydrazonate intermediates **9a**–**c** respectively. Structures **10a**–**c** are all supported by spectral and analytical data (*cf*. Scheme 2 and “Experimental”). A further evidence of the structures **10a**–**c** was deduced from alternative synthesis when the pyrazole **3a** reacted with ethyl chloroacetate to afford the thiazolyl-pyrazole derivative **11** which couples with the benzene diazonium chloride **12** to afford products which were found to be typically identical to **10a**. The identity was deduced from matching melting points, tlc and IR spectra.

The pyrazole derivative **3a** reacts also with the bromoacetyl derivatives **13a**–**c**, **16**, **18** and **20** to afford the corresponding thiazolyl-pyrazole derivatives **15a**–**c**, **17**, **19** and **21** respectively.

**Scheme 3:**
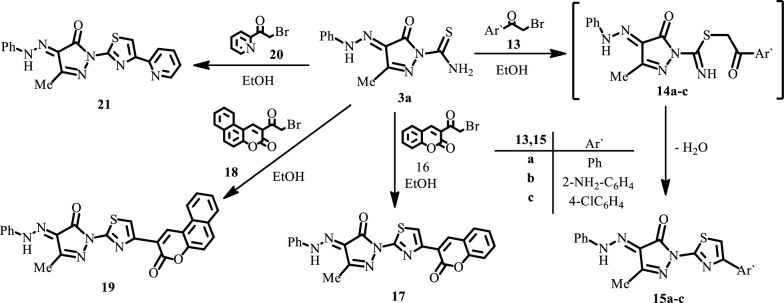
Synthesis of thiazole derivatives **15a**–**c**, **17**, **19** and **21**

Spectral data and elemental analyses of these products are in complete concurrence with the proposed structures (*cf*. Scheme 3 and “Experimental”).

### Molecular docking studies

The docking parameters were validated by redocking the native co-crystal ligand in order to determine the ability of MOE to reproduce the orientation and position of the native ligand in crystal structure, to ensure that the poses of the docked compounds represents a valid potential binding mode. The redocking of ligands with its targets revealed an RMSD ≤ 2.3 A° between the original ligand position and the docked poses which was RMSD = 1.3 A° this affirmed the ligands bound near the genuine pocket and adaptation of their objectives, this shows the dependability of conventions and parameters of docking. Molecular modeling studies were performed with MOE 2014, 0901, software available from Chemical Computing Group Inc., 1010 Sherbrooke Street West, Suite #910, Montreal, QC, Canada, H3A 2R7, 2014 [38].

#### Preparation of the ligand

The ligands (**3a**, **3b**, **6a**, **6b**, **6c**, **6d**, **10a**, **11**, **15a**, **15b**, **15c**, **17**, **19**, **21**) coordinates were built and modeled using the Chemsketch software (http://www.acdlabs.com/resources/freeware/).

Next, the right atom types (including hybridization states) and right bond types were characterized, hydrogen atoms were added, charges were relegated to each atom, lastly the structures were vitality limited by utilizing MOE program (MMFF94x, gradient: 0.01) [39]. The energies of ligand structures were minimized using the semiempirical AM1 strategy [40] with MOE program.

#### Selection of protein crystal structures

Crystallographic structures of EGFR with its Ligand is available in the Protein DataBank [41]. In this study, EGFR kinase crystal structure 1M17 is tested and selected for docking [42]. The errors of the protein were revised by the structure arrangement process in MOE. Reasonable protein structure is created by the task of hydrogen positions based on default rules. Water molecules contained have been expelled from the initially restored protein. Finally, partial charges were calculated by the Gasteiger methodology, and the active site of the ensemble has been characterized as the collection of residues within 10.0 A° of the bound inhibitor and comprised the union of all ligands of the ensemble. All atoms located less than 10.0 A° from any ligand atom were considered.

**Fig. 2:**
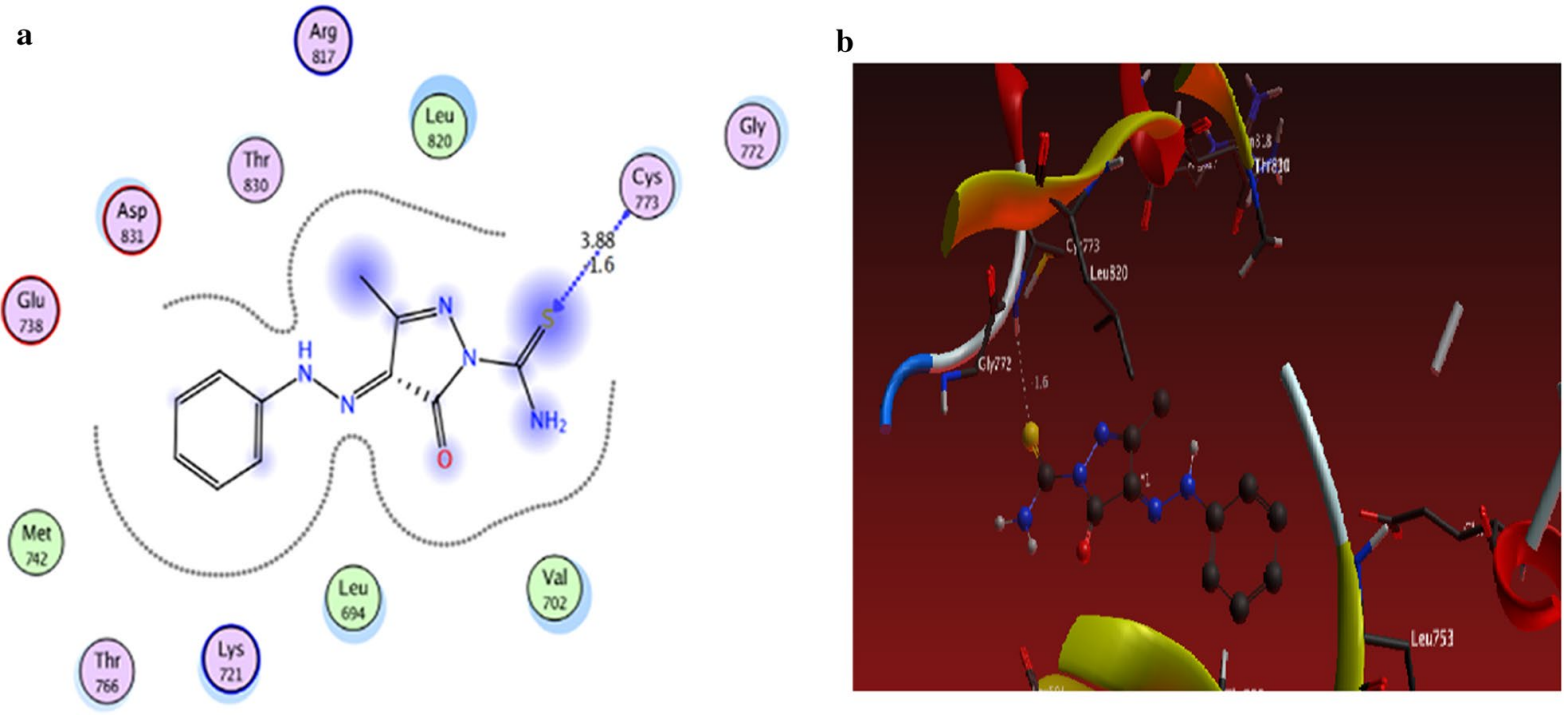
**a** 2D interaction between **3a** and amino acids of EGFR kinase, **b** 3D model of hydrogen bond interaction of **3a** with EGFR kinase

**Fig. 3:**
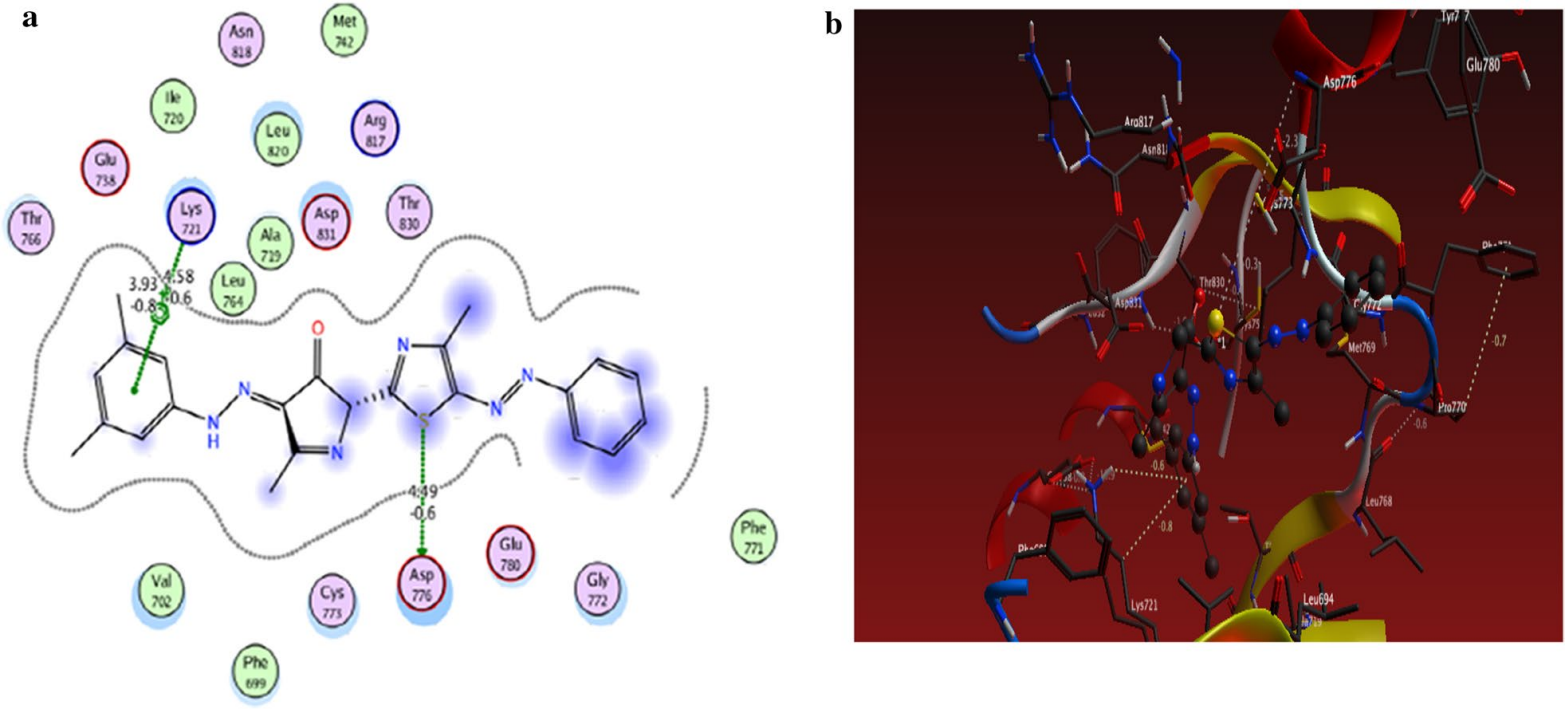
**a** 2D interaction between **6d** and amino acids of EGFR kinase, **b** 3D model of hydrogen bond interaction of **6d** with EGFR kinase

**Fig. 4:**
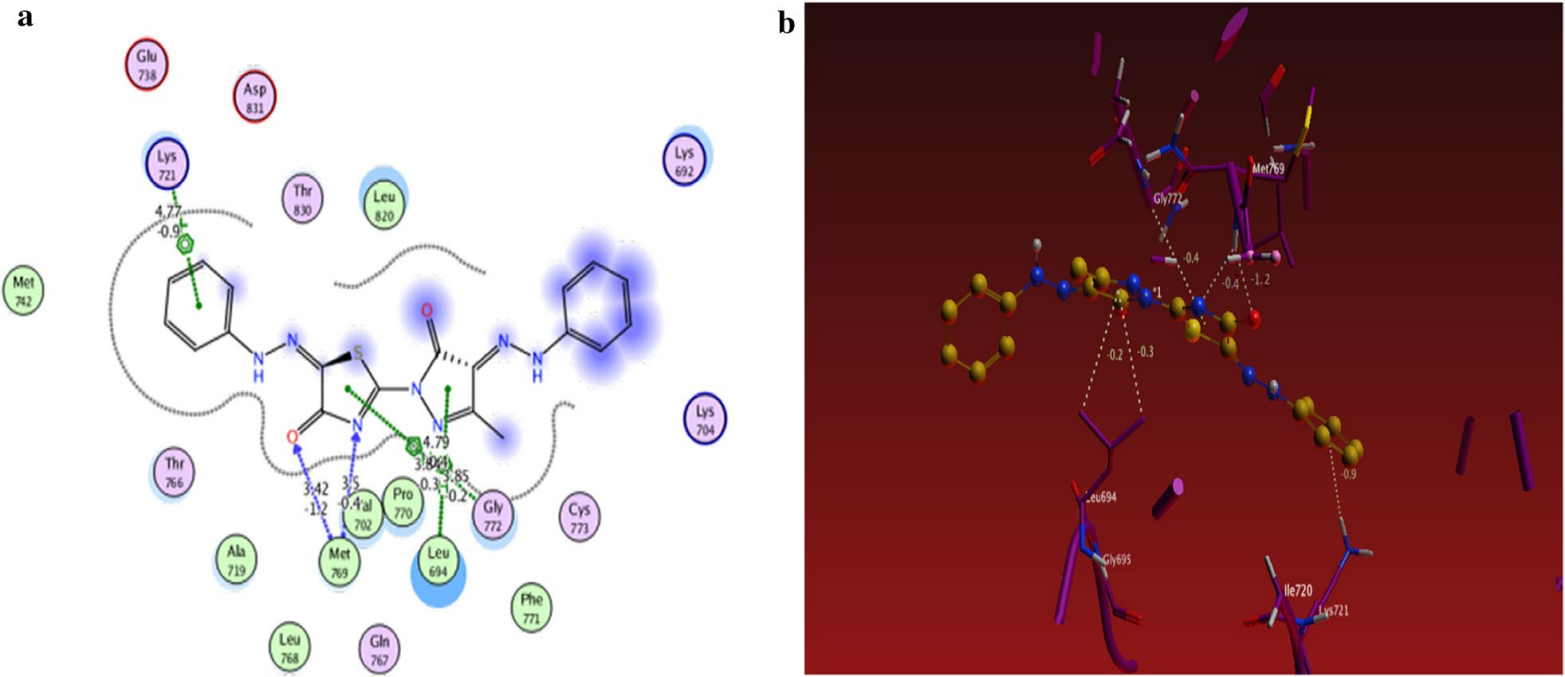
**a** 2D interaction between **10a** and amino acids Of EGFR kinase, **b** 3D model of hydrogen bond interaction of **10a** with EGFR kinase

**Fig. 5:**
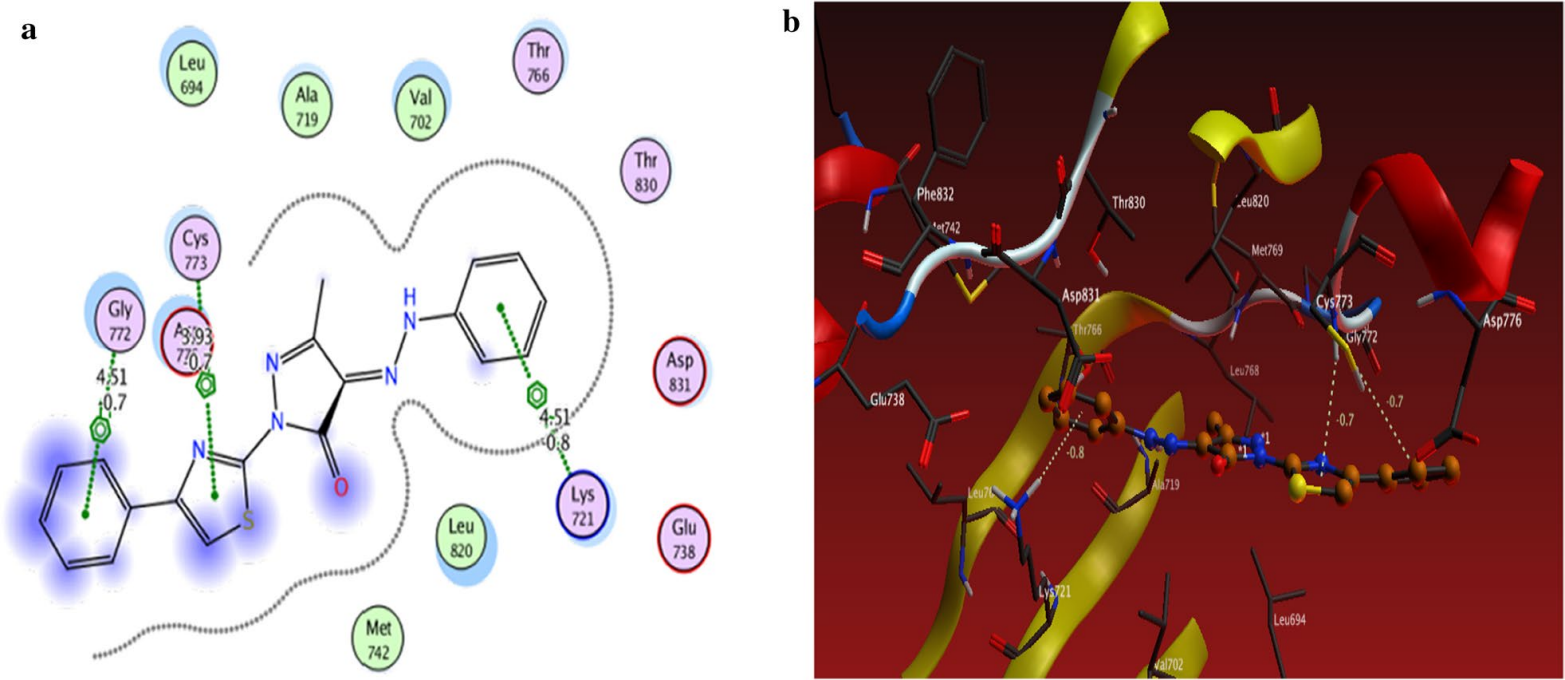
**a** 2D interaction between **15a** and amino acids of EGFR kinase, **b** 3D model of hydrogen bond interaction of **15a** with EGFR kinase

**Fig. 6:**
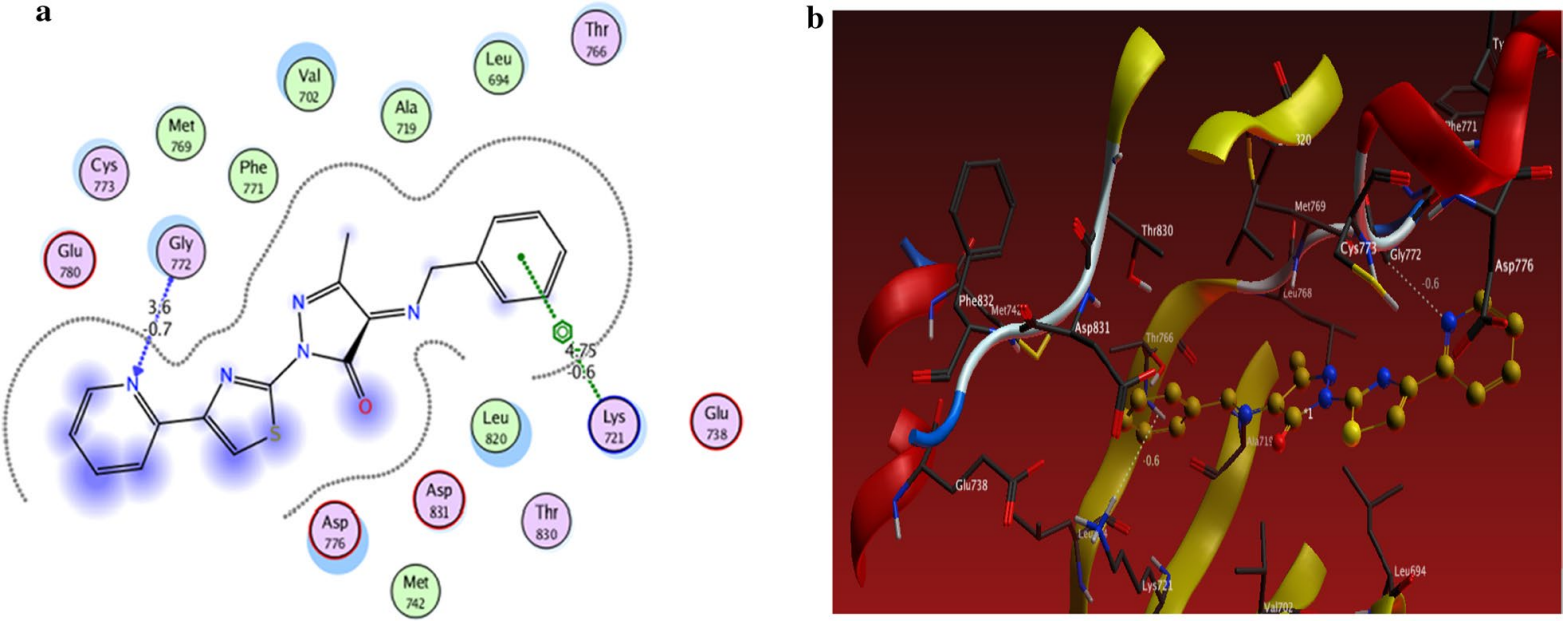
**a** 2D interaction between **21** and amino acids of EGFR kinase, **b** 3D model of hydrogen bond interaction of **21** with EGFR kinase

From the results obtained from docking studies as shown in Table 1, compounds **10a**, **6d**, **3a**, **21**, and **15a** were the most favorable compounds which meant by its lower binding energy (Binding energy = − 3.4, − 3.0, − 1.6, − 1.3 and − 2.2 kcal/mol, respectively), hydrogen bonding (number of H-bonds = 6, 3, 1, 2, and 3, respectively), and other hydrophobic interactions with the active site of the of EGFR kinase that might be one of the reasons for the good activities shown by these compounds in vitro studies (Figs. 2a, b, 3a, b, 4a, b, 5a, b, 6a, b).

**Table 1:** The interactions of the synthesized compounds with active sites of EGFR kinase

Compound	Binding energy (Kcal/mol)	No. of H-bonds	Length of H-bonds	Formed amino acids with H-bonds
**Reference legend: erlotinib** ([6,7-bis (2-methoxy-ethoxy)quinazoline-4-yl]-(3-ethynylphenyl)amine)	− 3.3	3	3.15 A 2.70 A 4.43 A	GLN 767 MET 769 GLY 772
**3a**	− 1.6	1	3.88 A	CYS 773
**3b**	− 0.3	1	4.26 A	VAL 702
**6a**	− 1.3	1	4.46 A	LYS 721
**6b**	− 1.3	1	4.83 A	LYS 721
**6c**	− 1.4	4	4.16 A 4.16 A 4.61 A 4.44 A	MET 742 HIS 781 LYS 721 GLY 772
**6d**	− 3.0	3	4.49 A 3.93 A 4.58 A	ASP 776 LYS 721 LYS 721
**10a**	− 3.4	6	3.50 A 3.42 A 3.84 A 3.85 A 4.77 A 4.79 A	MET 769 MET 769 LEU 694 LEU 694 LYS 721 GLY 772
**11**	− 1.1	3	4.05 A 4.33 A 3.38 A	MET 742 VAL 702 THR 766
**15a**	− 2.2	3	4.51 A 4.51 A 3.93 A	Lys 721 Gly 772 Cys 773
**15b**	− 0.8	1	3.75 A	Lys 721
**15c**	− 1.1	1	4.32 A	Lys 721
**17**	–	–	–	–
**19**	− 1.1	1	4.46 A	Lys 721
**21**	− 1.3	2	3.60 A 375 A	GLY 772 LYS 721

### Anticancer evaluation

**Fig. 7:**
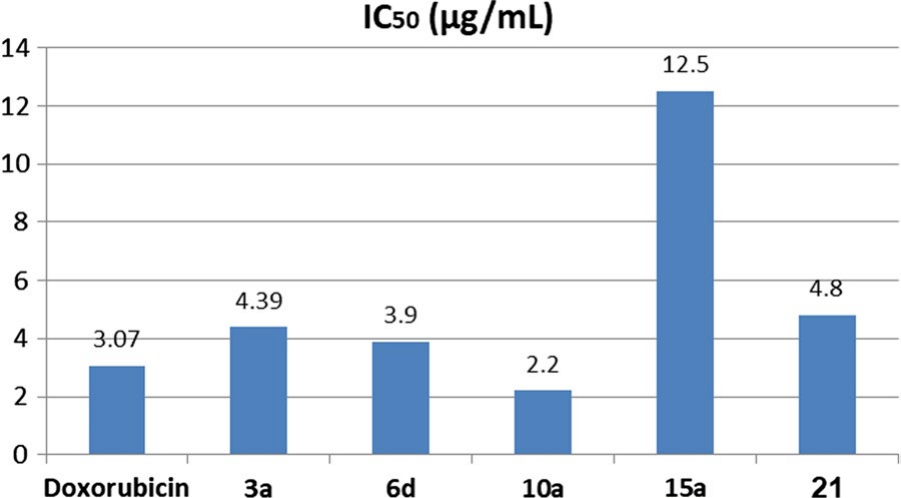
Cytotoxic activities of tested compounds against HepG-2

**Fig. 8:**
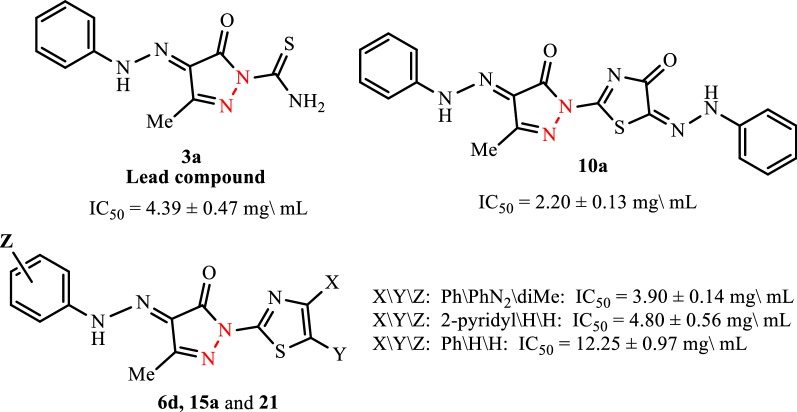
Cytotoxic activities of tested compounds against HepG-2

The cytotoxicity of five of the synthesized products **3a**, **6d**, **10a**, **15a** and **21** was evaluated against human liver carcinoma cell line (HepG-2) using the 3-(4,5-dimethylthiazol-2-yl)-2,5-diphenyl- tetrazolium bromide (MTT) assay and doxorubicin was used as a reference drug (IC_50_ value of doxorubicin = 3.07 ± 0.27 μg/mL). Data generated were used to plot a dose response curve of which the concentration of test compounds required to kill 50% of cell population (IC_50_) was determined. Cytotoxic activity was expressed as the mean IC_50_ of three independent experiments. The results are represented in Table 2 and Figs. 7 and 8.

**Table 2:** IC_50_ values of tested compounds ± standard deviation against HepG-2

Compound No.	IC_50_ (μg/mL)	Compound No.	IC_50_ (μg/mL)
**Doxorubicin**	3.07 ± 0.27	**10a**	2.20 ± 0.13
**3a**	4.39 ± 0.47	**15a**	12.5 ± 0.97
**6d**	3.90 ± 0.41	**21**	4.80 ± 0.56

#### Examination of the SAR leads to the following conclusions

- The descending order of activity of the new compounds was as follows: **10a** > **6d** > **3a** > **21** > **15a**.
- The phenylhydrazo-thiazolone **10a** showed higher antitumor inhibitory activities against HepG-2 cell lines (IC_50_ value of IC_50_ = 2.20 ± 0.13 μg/mL) than the standard doxorubicin drug (IC_50_ value of 3.07 ± 0.27 μg/mL). Accordingly, compound **10a** might be considered as a promising scaffold anti-liver cancer chemotherapeutic and deserves further optimization and in-depth biological studies.
- The 4-pyridyl-thiazole derivative **21** has higher antitumor activity than the 4-phenyl-thiazole derivative **15a**.
- Presence of two methyl groups (electron donating group) at position 3 and 5 of the phenyl ring in the arylhydrazo-pyrazolone moiety in compound **6d** increases its activity than the un-substituted compounds **6a**–**c**.

## Experimental

### Chemistry

#### General

Melting points were recorded in open capillaries using an electrothermal Gallenkamp apparatus and are uncorrected. Elemental analyses were carried out by the microanalytical center at Cairo University. The ^1^H NMR spectra were recorded on a Varian Mercury VXR-300 spectrometer and the chemical shifts were related to that of the solvent DMSO-*d*_*6*_. The mass spectra were recorded on a GCMSQ1000-EX Shimadzu spectrometers. The IR spectra were measured on a Pye-Unicam SP300 instrument.

#### Synthetic procedures

##### Synthesis of 3-methyl-5-oxo-4-(2-arylhydrazono)-4,5-dihydro-1*H*-pyrazole-1-carbothioamides **3a**,**b**

A catalytic amount of HCl was added to a mixture of ethyl 3-oxo-2-(2-arylhydrazono)butanoate derivatives **1a** or **1b** (10 mmol) and thiosemicarbazide (0.91 g, 10 mmol) in EtOH (40 mL) then the solution was refluxed for 6 h as indicated by TLC. The formed precipitate from reaction mixture was separated by filtration, washed with EtOH and recrystallized from dioxane to give pyrazole-1-carbothioamides **3a** and **3b,** respectively.

###### 3-Methyl-5-oxo-4-(2-phenylhydrazono)-4,5-dihydro-1*H*-pyrazole-1-carbothioamide (**3a**)

Orange solid, (78% yield), mp 230–232 °C (AcOH) (Lit. mp 224 °C [34]**)**; IR (KBr) *ν* = 3429–3263 (NH_2_ and NH), 3070, 2940 (C–H), 1684 (C=O), 1590 (C=N) cm^−1^; ^1^H NMR (DMSO-*d*_6_) *δ*: 2.25 (s, 3H, CH_3_), 7.22–7.65 (m, 5H, Ar–H), 8.91 (br s, 1H, NH), 9.43 (br s, 1H, NH), 13.06 (br s, 1H, SH). Anal. Calcd. For C_11_H_11_N_5_OS (261.30): C, 50.56; H, 4.24; N, 26.80. Found: C, 50.49; H, 4.13; N, 26.63%.

###### 4-(2-(3,5-Dimethylphenyl)hydrazono)-3-methyl-5-oxo-4,5-dihydro-1*H*-pyrazole-1-carbothioamide (**3b**)

Orange solid, (72% yield), mp 200–202 °C; IR (KBr) *ν* = 3423–3294 (NH_2_ and NH), 3093, 2970 (C–H), 1662 (C=O), 1607 (C=N) cm^−1^; ^1^H NMR (DMSO-*d*_6_) *δ*: 2.32 (s, 3H, CH_3_), 2.37 (s, 6H, 2CH_3_), 6.70 (s, 1H, Ar–H), 6.95 (s, 1H, Ar–H), 7.06 (s, 1H, Ar–H), 7.28 (br s, 2H, NH_2_), 12.87 (br s, 1H, NH); MS *m/z* (%): 289 (M^+^, 10), 230 (100), 214 (57), 140 (21), 105 (81), 91 (96), 77 (85). Anal. Calcd. For C_13_H_15_N_5_OS (289.36): C, 53.96; H, 5.23; N, 24.20. Found: C, 53.74; H, 5.14; N, 24.06%.

##### General method for synthesis of 5-(aryldiazenyl)thiazol-2-yl)-4-(2-phenylhydrazono)-1*H*-pyrazol-5(4*H*)-ones 6a-h and 5-oxo-4-(2-phenylhydrazono)-4,5-dihydro-1*H*-pyrazol-1-yl)-5-(2-phenylhydrazono)thiazol-4(5*H*)-one **11a**–**c**

Triethylamine (0.1 g, 1 mmol) was added to a cold mixture of 4,5-dihydro-1*H*-pyrazole-1-carbothioamides **3a** or **3b** (1 mmol) and appropriate hydrazonoyl halides **4** or **8** (1 mmol) in dioxane (15 mL). The formed solution was refluxed until complete reaction (3–6 h as monitored by TLC). Methanol was added to the residue formed after removing the excess solvent and the product separated was filtered, washed with methanol, dried and recrystallized from the proper solvent to give compounds **6a**–**h** and **10a**–**c**, respectively. The products **6a**–**h** and **10a**–**c** together with their physical constants are listed below.

###### 3-Methyl-1-(4-methyl-5-(phenyldiazenyl)thiazol-2-yl)-4-(2-phenylhydrazono)-1*H*-pyrazol-5(4*H*)-one (**6a**)

Orange solid, (72% yield), mp 215–217 °C (EtOH); IR (KBr) *ν* = 3428 (NH), 3050, 2980 (C–H), 1690 (C=O), 1597 (C=N) cm^−1^; ^1^H NMR (DMSO-*d*_6_) *δ*: 2.25 (s, 3H, CH_3_), 2.47 (s, 3H, CH_3_), 7.10–7.75 (m, 10H, Ar–H), 11.69 (br s, 1H, NH); ^13^C-NMR (DMSO-*d*_6_): *δ* 12.08, 15.13 (CH_3_), 117.17, 121.41, 123.63, 126.70, 126.83, 126.8, 130.07, 132.53, 129.8, 136.40, 139.79, 141.80, 150.16, 153.27, 157.16 (Ar–C and C=N), 177.39 (C=O); MS *m/z* (%): 403 (M^+^, 19), 330 (10), 202 (5), 180 (14), 118 (19), 104 (100), 91 (50), 77 (98), 65 (64). Anal. Calcd. For C_20_H_17_N_7_OS (403.46): C, 59.54; H, 4.25; N, 24.30. Found: C, 59.49; H, 4.21; N, 24.18%.

###### 3-Methyl-1-(4-methyl-5-(p-tolyldiazenyl)thiazol-2-yl)-4-(2-phenylhydrazono)-1*H*-pyrazol-5(4*H*)-one (**6b**)

Orange solid, (76% yield), mp 208–210 °C (EtOH); IR (KBr) *ν* = 3429 (NH), 3066, 2925 (C–H), 1658 (C=O), 1599 (C=N) cm^−1^; ^1^H NMR (DMSO-*d*_6_) *δ*: 2.13 (s, 3H, CH_3_), 2.25 (s, 3H, CH_3_), 2.47 (s, 3H, CH_3_), 7.14–7.51 (m, 9H, Ar–H), 11.53 (br s, 1H, NH); MS *m/z* (%): 417 (M^+^, 3), 261 (32), 202 (69), 125 (100), 93 (62), 77 (41), 65 (88). Anal. Calcd. For C_21_H_19_N_7_OS (417.49): C, 60.42; H, 4.59; N, 23.49. Found: C, 60.26; H, 4.52; N, 23.31%.

###### 1-(5-((3-Chlorophenyl)diazenyl)-4-methylthiazol-2-yl)-3-methyl-4-(2-phenylhydrazono)-1*H*-pyrazol-5(4*H*)-one (**6c**)

Orange solid, (76% yield), mp 218–220 °C (DMF); IR (KBr) *ν* = 3420 (NH), 3050, 2985 (C–H), 1669 (C=O), 1625 (C=N) cm^−1^; ^1^H NMR (DMSO-*d*_6_) *δ*: 2.25 (s, 3H, CH_3_), 2.48 (s, 3H, CH_3_), 7.14–7.65 (m, 9H, Ar–H), 11.53 (br s, 1H, NH); MS *m/z* (%): 437 (M^+^, 3), 261 (34), 202 (67), 125 (94), 93 (61), 77 (43), 65 (100). Anal. Calcd. For C_20_H_16_ClN_7_OS (437.91): C, 54.86; H, 3.68; N, 22.39. Found: C, 54.67; H, 3.49; N, 22.26%.

###### 4-(2-(3,5-Dimethylphenyl)hydrazono)-3-methyl-1-(4-methyl-5-(phenyldiazenyl)thiazol-2-yl)-1*H*-pyrazol-5(4*H*)-one (**6d**)

Yellow solid, (71% yield), mp 195–197 °C (EtOH); IR (KBr) *ν* = 3410 (NH), 3024, 2960 (C–H), 1660 (C=O), 1607 (C=N) cm^−1^; ^1^H NMR (DMSO-*d*_6_) *δ*: 2.32 (s, 3H, CH_3_), 2.36 (s, 6H, 2CH_3_), 2.43 (s, 3H, CH_3_), 6.85 (s, 1H, Ar–H), 6.92 (s, 1H, Ar–H), 7.06–7.28 (m, 6H, Ar–H), 11.50 (br s, 1H, NH); MS *m/z* (%): 431 (M^+^, 11), 430 (39), 325 (19), 230 (13), 105 (37), 91 (34), 77 (100), 67 (37). Anal. Calcd. For C_22_H_21_N_7_OS (431.51): C, 61.23; H, 4.91; N, 22.72. Found C, 61.15; H, 4.75; N, 22.65%.

###### 4-(2-(3,5-Dimethylphenyl)hydrazono)-3-methyl-1-(4-methyl-5-(p-tolyldiazenyl)thiazol-2-yl)-1*H*-pyrazol-5(4*H*)-one (**6e**)

Yellow solid, (75% yield), mp 181–183 °C (EtOH); IR (KBr) *ν* = 3413 (NH), 3040, 2958 (C–H), 1663 (C=O), 1606 (C=N) cm^−1^; ^1^H NMR (DMSO-*d*_6_) *δ*: 2.35 (s, 3H, CH_3_), 2.37 (s, 6H, 2CH_3_), 2.43 (s, 3H, CH_3_), 2.50 (s, 3H, CH_3_), 7.88–7.74 (m, 8H, Ar–H), 11.47 (br s, 1H, NH); MS *m/z* (%): 445 (M^+^, 36), 340 (15), 230 (31), 121 (26), 105 (45), 91 (100), 77 (48). Anal. Calcd. For C_23_H_23_N_7_OS (445.54): C, 62.00; H, 5.20; N, 22.01. Found: C, 61.92; H, 5.13; N, 21.83%.

###### 1-(5-((3-Chlorophenyl)diazenyl)-4-methylthiazol-2-yl)-4-(2-(3,5-dimethylphenyl)hydrazono)-3-methyl-1*H*-pyrazol-5(4*H*)-one (**6f**)

Yellow solid, (73% yield), mp 190–192 °C (EtOH); IR (KBr) *ν* = 3420 (NH), 3024, 2960 (C–H), 1661 (C=O), 1606 (C=N) cm^−1^; ^1^H NMR (DMSO-*d*_6_) *δ*: 2.35 (s, 3H, CH_3_), 2.37 (s, 6H, 2CH_3_), 2.42 (s, 3H, CH_3_), 6.88–7.28 (m, 8H, Ar–H), 11.40 (br s, 1H, NH); 465 (M^+^, 47), 360 (27), 230 (254), 152 (19), 125 (54), 105 (66), 77 (87), 67 (100). Anal. Calcd for C_22_H_20_ClN_7_OS (465.96): C, 56.71; H, 4.33; N, 21.04. Found: C, 56.55; H, 4.19; N, 20.85%.

###### 1-(5-((4-Chlorophenyl)diazenyl)-4-methylthiazol-2-yl)-4-(2-(3,5-dimethylphenyl)hydrazono)-3-methyl-1*H*-pyrazol-5(4*H*)-one (**6g**)

Yellow solid, (79% yield), mp 163–165 °C (EtOH); IR (KBr) *ν* = 3429 (NH), 3068, 2971 (C–H), 1657 (C=O), 1609 (C=N) cm^−1^; ^1^H NMR (DMSO-*d*_6_) *δ*: 2.25 (s, 3H, CH_3_), 2.36 (s, 6H, 2CH_3_), 2.56 (s, 3H, CH_3_), 6.82 (s, 1H, Ar–H), 7.02 (s, 1H, Ar–H), 7.28 (s, 1H, Ar–H), 7.45 (d, *J* = 8.8 Hz, 2H, Ar–H), 7.77 (d, *J* = 8.4 Hz, 2H, Ar–H), 11.88 (br s, 1H, NH); MS *m/z* (%): 465 (M^+^, 13), 332 (9), 253 (28), 230 (44), 154 (20), 125 (100), 77 (50), 67 (51). Anal. Calcd. for C_22_H_20_ClN_7_OS (465.96): C, 56.71; H, 4.33; N, 21.04. Found: C, 56.58; H, 4.14; N, 20.93%.

###### 1-(5-((2,4-Dichlorophenyl)diazenyl)-4-methylthiazol-2-yl)-4-(2-(3,5-dimethylphenyl) hydrazono)-3-methyl-1*H*-pyrazol-5(4*H*)-one (**6h**)

Yellowish-white solid, (83% yield), mp 270–272 °C (DMF); IR (KBr) *ν* = 3436 (NH), 3048, 2975 (C–H), 1654 (C=O), 1622(C=N) cm^−1^; ^1^H NMR (DMSO-*d*_6_) *δ*: 2.28 (s, 3H, CH_3_), 2.37 (s, 6H, 2CH_3_), 2.52 (s, 3H, CH_3_), 6.89–7.83 (m, 6H, Ar–H), 11.99 (br s, 1H, NH); MS *m/z* (%): 500 (M^+^, 42), 430 (39), 325 (19), 230 (13), 105 (37), 91 (34), 77 (100), 67 (37). Anal. Calcd. for C_22_H_19_Cl_2_N_7_OS (500.40): C, 52.80; H, 3.83; N, 19.59. Found: C, 52.63; H, 3.81; N, 19.46%.

##### Alternative synthesis of **6a**

To a solution of ethyl 3-oxo-2-(2-phenylhydrazono)butanoate (**1a**) (0.234 g, 1 mmol) in 2-propanol (10 mL), 2-hydrazinyl-4-methyl-5-(phenyldiazenyl)thiazole (**7**) (0.233 g, 1 mmol) was added. The mixture was refluxed for 3 h then cooled to room temperature. The solid precipitated was filtered off, washed with water, dried and recrystallized from EtOH to give the corresponding product, **6a** which were identical in all aspects (m.p., mixed m.p. and IR spectra) with those obtained from reaction of **3a** with **4a**.

###### 2-(3-Methyl-5-oxo-4-(2-phenylhydrazono)-4,5-dihydro-1*H*-pyrazol-1-yl)-5-(2-phenylhydra-zono)thiazol-4(5*H*)-one (**10a**)

Orange solid, (72% yield), mp 201–203 °C (EtOH); IR (KBr) *ν* = 3428, 3260 (2NH), 3062, 2926 (C–H), 1688, 1656 (2C=O), 1601 (C=N) cm^−1^; ^1^H NMR (DMSO-*d*_6_) *δ*: 2.30 (s, 3H, CH_3_), 7.14–7.65 (m, 10H, Ar–H), 9.43 (br s, 1H, NH), 11.53 (br s, 1H, NH); MS *m/z* (%): 405 (M^+^, 18), 261 (22), 202 (73), 125 (100), 93 (67), 65 (97), 51 (40). Anal. Calcd. For C_19_H_15_N_7_O_2_S (405.43): C, 56.29; H, 3.73; N, 24.18. Found: C, 56.15; H, 3.53; N, 24.07%.

###### 2-(3-Methyl-5-oxo-4-(2-phenylhydrazono)-4,5-dihydro-1*H*-pyrazol-1-yl)-5-(2-(m-tolyl)hydrazono)thiazol-4(5*H*)-one (**10b**)

Orange solid, (68% yield), mp 212–214 °C (EtOH); IR (KBr) *ν* = 3429, 3265 (2NH), 3015, 2935 (C–H), 1682, 1660 (2C=O), 1600 (C=N) cm^−1^; ^1^H NMR (DMSO-*d*_6_) *δ*: 2.25 (s, 3H, CH_3_), 2.47 (s, 3H, CH_3_), 7.24–7.67 (m, 9H, Ar–H), 9.43 (br s, 1H, NH), 11.46 (br s, 1H, NH); MS *m/z* (%): 419 (M^+^, 51), 261 (23), 202 (73), 125 (100), 93 (46), 65 (94), 51 (38). Anal. Calcd. For C_20_H_17_N_7_O_2_S (419.46): C, 57.27; H, 4.09; N, 23.37. Found: C, 57.09; H, 4.02; N, 23.22%.

###### 5-(2-(4-Chlorophenyl)hydrazono)-2-(3-methyl-5-oxo-4-(2-phenylhydrazono)-4,5-dihydro-1*H*-pyrazol-1-yl)thiazol-4(5*H*)-one (**10c**)

Orange solid, (73% yield), mp 150–152 °C (EtOH); IR (KBr) *ν* = 3428, 3287 (2NH), 3059, 2910 (C–H), 1686, 1654 (2C=O), 1603 (C=N) cm^−1^; ^1^H NMR (DMSO-*d*_6_) *δ*: 2.25 (s, 3H, CH_3_), 7.16–7.53 (m, 9H, Ar–H), 9.36 (br s, 1H, NH), 11.53 (br s, 1H, NH); MS *m/z* (%): 439 (M^+^, 2), 341 (27), 227 (12), 202 (60), 125 (100), 93 (61), 65 (96). Anal. Calcd. For C_19_H_14_ClN_7_O_2_S (439.88): C, 51.88; H, 3.21; N, 22.29. Found: C, 51.63; H, 3.28; N, 22.16%.

##### Alternate method for 10a

###### Synthesis of 2-(3-methyl-5-oxo-4-(2-phenylhydrazono)-4,5-dihydro-1*H*-pyrazol-1-yl)thiazol-4(5*H*)-one (**11**)

To a mixture of pyrazole-1-carbothioamide **3a** (2.61 g, 10 mmol) in ethanol (30 mL) containing anhydrous sodium acetate (3.3 g, 40 mmol), ethyl chloroacetate (1.22 g, l0 mmol) was added. The mixture was refluxed for 4–8 h (monitored by TLC), then left to cool. The solid product was filtered off, washed with ethanol and recrystalized from EtOH to afford the thiazolone derivative **11** as pale yellow solid (69% yield); mp 193–195 °C (EtOH); IR (KBr) *ν* = 3263 (NH), 3060, 2924 (C–H), 1688, 1659 (2C=O), 1583 (C=N) cm^−1^; ^1^H NMR (DMSO-*d*_6_) *δ*: 2.25 (s, 3H, CH_3_), 4.14 (s, 2H, CH_2_), 7.17–7.63 (m, 5H, Ar–H), 9.45 (br s, 1H, NH); ^13^C-NMR (DMSO-*d*_6_): *δ* 12.07 (CH*3*), 31.52 (CH_2_), 117.12, 126.67, 126.78, 130.03, 141.75, 150.14, 157.16 (Ar–C and C=N), 172.47, 177.40 (C=O); MS, *m/z* (%) 301 (M^+^, 64), 261 (56), 202 (73), 125 (100), 93 (79), 77 (53), 65 (84). Anal. Calcd for C_13_H_11_N_5_O_2_S (301.32): C, 51.82; H, 3.68; N, 23.24. Found C, 51.71; H, 3.60; N, 23.08%.

##### Coupling of thiazolone derivative **11** with benzenediazonium chloride **12**

A cold solution of benzenediazonium chloride **12** was added portionwise to a cold solution of **11** (0.301 g, 1 mmol) in pyridine (20 mL). After complete addition of the diazonium salt, the solid that separated was filtered off, washed with water and finally recrystallized from EtOH to give a product proved to be identical in all respects (IR spectra, mp and mixed mp) with compound **10a** which was resulted from reaction of **3a** with **8a**.

##### Synthesis of 3-methyl-4-(2-phenylhydrazono)-1-(4-aryl(heteryl)thiazol-2-yl)-1*H*-pyrazol-5(4*H*)-ones **15a**–**c**, **17**, **19** and **21**

General procedure: An ethanolic soluton of 3-methyl-5-oxo-4-(2-phenylhydrazono)-4,5-dihydro-1*H*-pyrazole-1-carbothioamide (**3a**) (0.261 g, 1 mmol) and α-bromoacetyl derivatives **13a**–**c** or **16** or **18** or **20** (1 mmol) was refluxed for 4–8 h, then left to cool. The solid product was filtered off, washed with ethanol and recrystallized from dioxane to afford the thiazole derivatives **15a**–**c**, **17**, **19** and **21**, respectively.

###### 3-Methyl-4-(2-phenylhydrazono)-1-(4-phenylthiazol-2-yl)-1*H*-pyrazol-5(4*H*)-one (**15a**)

Orange solid (70% yield); mp 277–279 °C (DMF); IR (KBr): *v*/cm^−1^ 3429 (NH), 3061, 2958 (C–H), 1685 (C=O), 1583 (C=N); ^1^H-NMR (DMSO-*d*_6_): *δ* 2.44 (s, 3H, CH_3_), 7.28 (s, 1H, thiazole H5), 7.30–7.51 (m, 10H, Ar–H), 9.06 (br s, 1H, NH); ^13^C-NMR (DMSO-*d*_6_): *δ* 12.08 (CH_3_), 117.15, 123.01, 126.68, 126.81, 129.11, 130.06, 134.50, 141.78, 148.00, 150.15, 152.04, 155.15, 157.16, (Ar–C and C=N), 171.40 (C=O); MS *m/z* (%): 361 (M^+^, 31), 284 (24), 202 (89), 125 (100), 93 (83), 77 (62). Anal. calcd for C_19_H_15_N_5_OS (361.42): C, 63.14; H, 4.18; N, 19.38. Found: C, 63.03; H, 4.11; N, 19.25%.

###### 1-(4-(4-Aminophenyl)thiazol-2-yl)-3-methyl-4-(2-phenylhydrazono)-1*H*-pyrazol-5(4*H*)-one (**15b**)

Orange solid (75% yield); mp 230–232 °C (DMF/EtOH); IR (KBr): *v*/cm^−1^ 3428–3260 (NH and NH_2_), 3069, 2959 (C–H), 1686 (C=O), 1582 (C=N); ^1^H-NMR (DMSO-*d*_6_): *δ* 2.42 (s, 3H, CH_3_), 7.28 (s, 1H, thiazole H5), 7.07 (br s, 2H, NH_2_), 7.30–7.54 (m, 9H, Ar–H), 9.06 (br s, 1H, NH); MS *m/z* (%): 376 (M^+^, 9), 261 (68), 202 (100), 125 (94), 93 (76), 77 (40), 65 (77).Anal. calcd for C_19_H_16_N_6_OS (376.43): C, 60.62; H, 4.28; N, 22.33. Found: C, 63.55; H, 4.14; N, 22.16%.

###### 1-(4-(4-Chlorophenyl)thiazol-2-yl)-3-methyl-4-(2-phenylhydrazono)-1*H*-pyrazol-5(4*H*)-one (**15c**)

Orange solid (78% yield); mp 215–217 °C (DMF/EtOH); IR (KBr): *v*/cm^−1^ 3428 (NH), 3069, 2959 (C–H), 1686 (C=O), 1582 (C=N); ^1^H-NMR (DMSO-*d*_6_): *δ* 2.43 (s, 3H, CH_3_), 7.28 (s, 1H, thiazole H5), 7.39 (d, *J* = 8.4 Hz, 2H, Ar–H), 7.47–7.49 (m, 5H, Ar–H), 7.92 (d, *J* = 8.4 Hz, 2H, Ar–H), 9.05 (br s, 1H, NH); MS *m/z* (%): 395 (M^+^, 80), 318 (66), 261 (52), 202 (57), 125 (76), 93 (41), 77 (58), 67 (100). Anal. calcd for C_19_H_14_ClN_5_OS (395.87): C, 57.65; H, 3.56; N, 17.69. Found: C, 57.39; H, 3.38; N, 17.60%.

###### 3-Methyl-1-(4-(2-oxo-2*H*-chromen-3-yl)thiazol-2-yl)-4-(2-phenylhydrazono)-1*H*-pyrazol-5(4*H*)-one (**17**)

Orange solid (75% yield); mp 239–241 °C (DMF); IR (KBr): *v*/cm^−1^ 3412 (NH), 3054, 2920 (C–H), 1715, 1689 (2C=O), 1596 (C=N); ^1^H-NMR (DMSO-*d*_6_): *δ* 2.42 (s, 3H, CH_3_), 7.28 (s, 1H, thiazole H5), 7.30–7.69 (m, 9H, Ar–H), 8.35 (s, 1H, Coumarine H4), 8.87 (br s, 1H, NH); MS *m/z* (%): 429 (M^+^, 100), 401 (27), 352 (53), 271 (19), 255 (50), 171 (9), 77 (37), 67 (80). Anal. calcd for C_22_H_15_N_5_O_3_S (429.45): C, 61.53; H, 3.52; N, 16.31. Found: C, 61.42; H, 3.37; N, 16.22%.

###### 3-Methyl-1-(4-(3-oxo-3*H*-benzo[f]chromen-2-yl)thiazol-2-yl)-4-(2-phenylhydrazono)-1*H*-pyrazol-5(4*H*)-one (**19**)

Orange solid (78% yield); mp 280–282 °C (DMF); IR (KBr): *v*/cm^−1^ 3429 (NH), 3057, 2957 (C–H), 1722, 1675 (2C=O), 1629 (C=N); ^1^H-NMR (DMSO-*d*_6_): *δ* 2.41 (s, 3H, CH_3_), 7.28 (s, 1H, thiazole H5), 7.46–7.50 (m, 6H, Ar–H), 7.61 (d, *J* = 7.2 Hz, 1H, Ar–H), 7.75 (t, *J* = 8.00 Hz, 1H, Ar–H), 7.92 (d, *J* = 5.6 Hz, 1H, Ar–H), 8.00 (d, *J* = 8.0 Hz, 1H, Ar–H), 8.42 (s, 1H, Coumarine H4), 8.61 (d, *J* = 8.4 Hz, 1H, Ar–H), 9.06 (br s, 1H, NH); MS *m/z* (%): 479 (M^+^, 100), 451 (27), 305 (48), 222 (9), 139 (7), 77 (39), 67 (80). Anal. calcd for C_26_H_17_N_5_O_3_S (479.51): C, 65.12; H, 3.57; N, 14.61. Found: C, 65.02; H, 3.41; N, 14.39%.

###### 3-Methyl-4-(2-phenylhydrazono)-1-(4-(pyridin-2-yl)thiazol-2-yl)-1*H*-pyrazol-5(4*H*)-one (**21**)

Orange solid (73% yield); mp 240–242 °C (DMF); IR (KBr): *v*/cm^−1^ 3428 (NH), 3069, 2959 (C–H), 1686 (C=O), 1627 (C=N); ^1^H-NMR (DMSO-*d*_6_): *δ* 2.42 (s, 3H, CH_3_), 7.28 (s, 1H, thiazole H5), 7.30–7.78 (m, 7H, Ar–H), 8.40 (d, 1H, Ar–H), 8.72 (d, 1H, Ar–H), 9.06 (br s, 1H, NH); MS *m/z* (%): 362 (M^+^, 8), 261 (51), 202 (77), 125 (100), 93 (78), 77 (45), 65 (19). Anal. calcd for C_18_H_14_N_6_OS (362.41): C, 59.65; H, 3.89; N, 23.19. Found: C, 59.42; H, 3.69; N, 23.11%.

### Molecular modeling

Docking Study was performed using the MOE 2014.09 software. Regularization and optimization for protein and ligand were performed. Each docked compound was assigned a score according to its fit in the ligand binding pocket (LBP) and its binding mod.

### Cytotoxic activity

In this study, the newly synthesized compounds were subjected to cytotoxic evaluation on human tumour cell line [43].

#### Materials and methods

##### Chemicals

All chemicals used in this study are of high analytical grade. They were obtained from (either Sigma-Alderich or Biorad).

##### Human tumor cell lines

The tumour cell lines were obtained frozen in liquid nitrogen (− 180 °C) from the American Type Culture Collection (ATCC) and was maintained at the National Cancer Institute, Cairo, Egypt, by serial subculturing.

##### Measurement of potential cytotoxic activity

The cytotoxic activity was measured in vitro on human cancer cell line (HEPG2) using Sulforhodamine-B stain (SRB) assay.

Cells were plated in 96 multi well plates for 24 h before treatment with the compounds to allow attachment of the cells to the wall of the plate.

- Different concentrations of the compound under test (0, 6.25, 12.5, 25, 50 and 100 µg/mL) were added to the cell monolayer. Triplicate wells were prepared for each individual dose.
- Monolayer cells were incubated with the compounds for 48 h at 37 °C and in atmosphere of 5% CO_2_.
- After 48 h cell was fixed, washed and stained with Sulforhodamine B stain.
- Excess stain was washed with acetic acid and attached stain was recovered with Tris EDTA buffer.
- Colour intensity was measured in an ELISA reader.
- The relation between surviving fraction and drug concentration was plotted and IC_50_ (the concentration required for 50% inhibition of cell viability) was calculated for each compound by Sigmaplot software.

## Conclusions

We portrayed a convenient and efficient synthesis of numerous diversely substituted thiazolyl-pyrazole derivatives from cheap laboratory accessible starting materials. Since a variety of thiazolyl-pyrazole derivatives **6a**–**h**, **10a**–**c**, **15a**–**c**, **17**, **19** and **21** were synthesized from reaction of 3-methyl-5-oxo-4-(2-arylhydrazono)-4,5-dihydro-1*H*-pyrazole-1-carbothioamides **3a**,**b** with a diversity hydrazonoyl chlorides as well as bromoacetyl derivatives. Simple synthetic routes were pursued and no risky solvents, catalysts or substantial metals were included. Moreover, the computational studies were carried out for all the products and the results revealed that four new compounds showed promising binding affinities against EGFR. The cytotoxicity of the potent products was tested against HepG-2 using Doxorubicin standard drug. There was an agreement between the benefits of binding affinities and the data obtained from the practical anticancer screening of the tested compounds.

## Data Availability

The datasets and samples of the compounds used during the current study are available from the corresponding author on reasonable request.

## Abbreviations

TEA: triethylamine
TLC: thin layer chromatography
HepG2: human hepatocellular carcinoma
EtOH: ethanol
m.p.: melting point
IR: infra-red
ATCC: American Type Culture Collection
EGFR: the epidermal growth factor receptor kinase
